# LASS2 impairs proliferation of glioma stem cells and migration and invasion of glioma cells mainly via inhibition of EMT and apoptosis promotion

**DOI:** 10.7150/jca.71256

**Published:** 2022-04-18

**Authors:** Wei-Jiang Zhao, Yi-Pu Fan, Guan-Yong Ou, Xin-Yu Qiao

**Affiliations:** 1 Cell Biology Department, Wuxi School of Medicine, Jiangnan University, Wuxi 214122, Jiangsu, P.R. China.; 2 Center for Neuroscience, Shantou University Medical College, Shantou 515041, Guangdong, P.R. China.

**Keywords:** glioma, glioblastoma, glioma stem cells, LASS2, epithelial-mesenchymal transition (EMT), migration, invasion

## Abstract

LAG1 longevity assurance homolog 2 (LASS2), a highly conserved transmembrane protein, has been reported in several cancer types. However, the roles of LASS2 in glioma biology remain elusive. In the present study, we investigated the expression of LAAS2 in human glioma tissues and the effects of LASS2 on glioma stem cell (GSC) proliferation. Roles of LASS2 in glioma cell migration and invasion were also researched both *in vitro* and *in vivo*. Our results demonstrated that the level of LASS2 is gradually reduced with the increase of glioma grade. The level of LASS2 is significantly lower in GSCs than in non GSCs, whereas *LASS2* overexpression reduced the sphere formation and promoted the differentiation of CD133^+^ glioblastoma cells, as was indicated by reduced levels of CD133 and Nestin. In addition, *LASS2* overexpression significantly reduced colony formation, migration, and invasion of glioma cells by promoting tumor cell apoptosis and inhibiting epithelial-mesenchymal transition (EMT). Overexpression of *LASS2* inhibited U-87 MG cell-derived glioma xenograft growth in nude mice in a manner similar to *in vitro*. Our findings indicate that LASS2 can function as a suppressor of glioma growth, suggesting that modulation of *LASS2* expression may contribute to a novel strategy for the management of glioma via inhibition of GSCs.

## Introduction

Malignant glioma is the most common central nervous system tumor and is highly lethal. It accounts for approximately 70% of all human primary brain tumors in adults 1. The 5-year survival rate of patients with high-grade gliomas is less than 5% 2. At present, malignant gliomas are highly associated with poor prognosis and high recurrence due to their low sensitivity to radiotherapy and chemotherapy 3. Thus, it is urgently needed to identify the mechanisms underlying the development and progression of gliomas.

Accumulated evidence indicates that glioma stem cells (GSCs), a rare subpopulation characterized by self-renewal and tumorigenicity, are responsible for the malignant development of gliomas 4, 5. Efficient and reliable isolation of GSCs may allow an improved interpretation of the biological characteristics of GSCs and lead to the emergence of novel therapeutic regimens 6. However, the underlying molecular mechanisms responsible for the contribution of GSCs to glioma initiation and development remain unelucidated.

*Homo sapiens* longevity assurance homolog 2 of yeast *LAG1* (*LASS2*), also named *TMSG1* (tumor metastasis suppressor gene) and *CERS2* (ceramide synthase 2), was first identified from a human liver cDNA library 7. *LASS2* is a member of the *LASS* family, which is composed of *LASS1-6* 8. It is highly expressed in nonmetastatic tumors, negatively correlated with metastasis 9. In addition, it negatively regulates the metastatic potential of breast cancer cells 7. These combined data suggest that the LASS2 protein plays pivotal roles in suppressing metastasis and invasion of most tumor types. However, the function of LASS2 in glioma remains unknown. We here studied the role of LASS2 in glioma biology both *in vitro* and *in vivo*.

Our main findings include: 1) LASS2 is lowly expressed in high-grade gliomas and GSCs; 2) *LASS2* overexpression reduced the sphere formation and promoted the differentiation of glioma stem cells; 3) *LASS2* overexpression significantly reduced colony formation, migration, and invasion of glioma cells, and 4) Overexpression of *LASS2* inhibited U-87 MG cell-derived glioma xenograft growth in nude mice. Our findings suggest that LASS2 can function as a suppressor of glioma growth, and inhibition of GSCs via modulation of *LASS2* expression may represent a novel strategy for the management of glioma.

## Materials and Methods

### Animals

Four-week-old male nude mice were purchased from the Model Animal Research Center of Nanjing University and maintained under SPF (specific pathogen-free) conditions according to the NIH guidelines at the Animal Facility of Shantou University Medical College. The mice were housed in a temperature (22 ± 1 °C)- and humidity (55 ± 5%)-controlled room, with free access to water and food. All the animal experiments were conducted with the approval of the Institutional Animal Care and Use Committee of Shantou University Medical College (Approval No. SUMC 2018-119).

### Cells, human glioma tissue microarray, and plasmids

U251 (Cat. No. CL-0237) human glioblastoma and U-87 MG (Cat. No. CL-0238) human glioblastoma cell lines were provided by Procell Life Science & Technology Co., Ltd (Wuhan, China) and were cultured in DMEM (Dulbecco's modified Eagle's medium)/low glucose (SH30021.01, Thermo Scientific HyClone, Beijing, China) supplemented with 10% FBS (fetal bovine serum; Sijiqing Biotech, Hangzhou, China) and 50 U/ml penicillin/streptomycin solution (Solarbio Biotech, Beijing, China). Both U251 and U-87 MG cell lines have been typically used to investigate development and treatment mechanisms of glioma/glioblastoma, with U-87 MG cells showing more malignancy potency. Both cell lines were authenticated at Procell by STR profiling. No contamination of HIV-1, HBV, HCV, mycoplasma, bacteria, and yeast has been identified by the company. A human glioma microarray containing glioma tissues graded from I to IV according to the malignancy degree (pilocytic astrocytoma (WHO grade I), well-differentiated astrocytoma (WHO grade II), anaplastic astrocytoma (WHO grade III), and glioblastoma (WHO grade IV)) was purchased from Alenabio Biotechnology (GL 2083, Xian, Shaanxi, China). Plasmids pLV-Vec (pLV [Exp] -EGFP: T2A: Puro-EF1A), pLV-LASS2 (pLV [Exp] -EGFP: T2A: Puro-EF1A>hCERS2[NM_022075.4]) and pLV-shLASS2 (pLV [shRNA] -EGFP:T2A:Puro-U6>hCERS2[shRNA#1]) were purchased from VectorBuilder (Guangzhou, China).

### Isolation of CD133^+^ GSCs and CD133^-^ non-GSCs from U-87 MG cells

Approximately 1 × 10^7^ cells of either the U-87 MG cell line or the U251 cell line were collected for analysis of CD133^+^ cells by flow cytometry. After washing with PBS three times, cells were suspended in 100 μl of flow buffer (PBS with 2 mM EDTA and 0.5% BSA; pH 7.2) containing a PE-conjugated mouse anti-human CD133 antibody (1:10; 130-090-853; Miltenyi, Germany). The cells were incubated in the dark for 30 min at 4 °C, washed with flow buffer, and centrifuged at 1300 rpm for 5 min. The cells were then resuspended in a flow buffer for subsequent analysis. In each analysis, a total of 1 × 10^4^ cells were harvested to separate the CD133^+^ and CD133^-^ cell subsets. Flow cytometric analysis and cell sorting were performed with a BD Accuri C6 flow cytometer and a BD FACS Aria II cell sorter (Becton and Dickinson Company, Franklin Lakes, NJ, USA), respectively. CD133^+^ GSCs sorted from U-87 MG cells were maintained in DMEM-F12 medium containing 20 ng/ml human recombinant epidermal growth factor (EGF) (Invitrogen), 20 ng/ml human recombinant basic fibroblast growth factor (bFGF) (Invitrogen), 2% B27 (Invitrogen), and 1% penicillin/streptomycin solution (Solarbio Biotech Corp., Beijing, China). Then, 10^6^ cells were harvested and stained under conditions identical to those described above.

### Wound healing assay

U251 and U-87 MG cells stably transfected with either pLV-LASS2 (2 µg; VectorBuilder, Guangzhou, China) or pLV-Vec (2 µg; VectorBuilder, Guangzhou, China) were seeded in 96-well plates (5 × 10^4^ cells/well) in DMEM culture medium supplemented with 10% FBS. A 10 μl plastic pipette tip was used to create uniform scratch wounds in the cell monolayers. After 0, 12, and 24 h of incubation at 37 °C, the wound widths were examined under a phase-contrast microscope (IX51, Olympus, Tokyo, Japan) at 10× magnification. At least three random fields were photographed, and the cell migration ability was evaluated by assessing the gap closure distance.

### Colony formation assay

U251 and U-87 MG cells were stably transfected with pLV-LASS2 (2 μg; VectorBuilder, Guangzhou, China) or pLV-Vec (2 μg; VectorBuilder, Guangzhou, China), and CD133^-^ or CD133^+^ U-87 MG cells were seeded in 24-well plates at 1500 cells/well and incubated for 10 days to evaluate colony formation. Colonies were fixed for 20 min with 4% PFA, stained with 0.1% crystal violet solution, and eventually visualized under a phase-contrast microscope (IX51, Olympus) at 4× magnification. Colony formation was defined as the cumulative growth of more than 50 cells.

### Transwell assay

U251 and U-87 MG cells were stably transfected with pLV-LASS2 (2 μg; VectorBuilder, Guangzhou, China) or pLV-Vec (2 μg; VectorBuilder, Guangzhou, China) and were then seeded on polycarbonate membrane inserts with 8-μm pores (with the underside precoated with fibronectin) in a transwell chamber (Costar, Corning, NY, USA) or on the upper side of membranes precoated with Matrigel (BD, Franklin Lakes, NJ, USA). Then, 500 μl of DMEM culture medium supplemented with 10% FBS was added to the lower chambers as a chemoattractant. After incubation for 12 h, the inserts were washed with PBS 3 times, and cells on the top surface were gently removed with a cotton swab. The cells attached to the underside were fixed for 20 min with 4% PFA, followed by staining with 0.1% crystal violet solution. Retained cells were counted under a phase-contrast microscope (IX51, Olympus). All assays were independently repeated at least three times.

### RNA sequencing and data analysis

Total RNA was prepared using a simple Total RNA Kit (DP419; Tiangen, Beijing, China). High-throughput RNA sequencing and analysis were performed on a BGISEQ 500 sequencer (Beijing Genomics Institute (BGI), Shenzhen, China).

### Neurosphere formation assay

CD133^+^ and CD133^-^ or CD133^+^-pLV-LASS2 and CD133^+^-pLV-Vec cells were plated in 24-well plates at the indicated numbers of cells per well. All cells were maintained in DMEM-F12 medium supplemented with 20 ng/ml EGF, 20 ng/ml bFGF, 2% B27 supplement (Invitrogen), and 1% penicillin/streptomycin solution (Solarbio Biotech Corp., Beijing, China). The number of neurospheres in each well was quantified. Neurospheres were then dissociated into single cells and replated in 24-well plates at the indicated number of cells per well to generate a second generation of neurospheres.

### Immunofluorescence staining

For tissue immunofluorescence staining, the deparaffinized human glioma tissue microarray section was rehydrated and antigen retrieval was performed. Then, the section was blocked with 10% normal donkey serum (NDS) and incubated with a mixture of primary mouse anti-CD133 (1:200; 66666-1-Ig, Proteintech, Wuhan, China) and rabbit anti-LASS2 (1:200; 20344-1-AP; Proteintech) antibodies overnight at 4 °C. For cell immunofluorescence staining, CD133^+^ cells were seeded on coverslips pretreated with poly-L-lysine (Sigma-Aldrich, St. Louis, MO, USA) overnight. Then, the cells were fixed and incubated with antibodies against CD133 (1:50; 18470-1-AP, Proteintech) and Nestin (1:50; 66259-1-Ig, Proteintech) overnight at 4 °C. In addition, U251 and U-87 MG cells stably transfected with pLV-LASS2 (2 μg; VectorBuilder, Guangzhou, China) or pLV-Vec (2 µg; VectorBuilder, Guangzhou, China) were seeded on coverslips in a 24-well plate at a density of 5 × 10^4^ cells/well. Cells were then fixed and incubated with an anti-MMP9 antibody (1:100; sc-6840, Santa Cruz) or an anti-SPHK1 antibody (1:100; 10670-1-AP, Proteintech) overnight at 4 °C. After washing in PBS three times for 5 min each, either tissue microarray section or cell samples were incubated at room temperature with a DyLight^TM^ 488-conjugated goat anti-mouse secondary antibody (1:500; Jackson ImmunoResearch, Inc., West Grove, PA, USA), and DyLight^TM^ 594-conjugated goat anti-rabbit secondary antibody (1:500; Jackson ImmunoResearch, Inc.) for 60 min. Samples were then stained with 4',6-diamidino-2-phenylindole (DAPI), followed by mounting with an anti-fade mounting solution (Beyotime). Images were finally acquired using an Olympus confocal system (FV-1000, Olympus, Japan).

### Nude mouse xenograft model

To evaluate expression differences between non-GSCs and GSCs *in vivo*, CD133^-^ (5 × 10^5^ cells/mouse) and CD133^+^ (5 × 10^5^ cells/mouse) cells were subcutaneously injected into mice (n=3 mice per group). To evaluate the effect of LASS2 on the growth of glioma *in vivo*, U-87 MG-pLV-LASS2 and U-87 MG-pLV-Vec cells (5 × 10^5^ cells/mouse) were injected subcutaneously into the flanks of mice (n=5 mice per group). When the tumors were palpable, tumor growth was monitored using a Vernier caliper (BST-01601; BESTIR, Foshan, China) on days 7, 10, 14, 16, 18, and 21. The volume of glioma xenografts was estimated using the formula L × S^2^/2, where L is the longest diameter and S is the shortest diameter. After mice were euthanized, the tumors were collected and weighed using an electronic balance (ME104; Mettler Toledo, Shanghai, China). All animal experiments were performed in accordance with the NIH guidelines and the guidelines of the Institutional Animal Care and Use Committee of Shantou University Medical College (ethics approval number: SUMC2018-119, permission date: 30 May 2018).

### Western blot analysis

Cells or tissues were lysed in RIPA buffer (Solarbio Biotech, Beijing, China) supplemented with PMSF (1:200; Solarbio Biotech). After centrifugation at 14,000 ×*g* for 15 min at 4 °C, the supernatants of the cell lysates were collected. Equivalent quantities of either cell or tissue lysates were first boiled at 100 °C in 20% protein loading buffer (0.125 mol/l Tris-HCl (pH 6.8), 20% glycerol, 10% sodium dodecyl sulfate, 0.1% bromophenol blue, and 5% β-mercaptoethanol). Proteins were subsequently resolved by 10% SDS-PAGE (sodium dodecyl sulfate-polyacrylamide) gel electrophoresis and immediately transferred to a PVDF (polyvinylidene difluoride) membrane (Millipore, Billerica, MA, USA). Then, 5% BSA diluted in Tris-buffered saline buffer containing 0.05% Tween 20 (TBST, pH 7.4) was used to block nonspecific protein binding sites at room temperature for 1 h. The membranes were incubated with antibodies specific for Bcl-2 (C-2; 1:1000; sc-7382), Bax (P-19; 1:1000; sc-526), MMP2 (K-20; 1:1000; sc-8835), MMP9 (C-20; 1:1000; sc-6840), and GAPDH (2E3-2E10; 1:1000; sc-293335) (all obtained from Santa Cruz Biotechnology, Santa Cruz, CA, USA) and other antibodies specific for both pro- and cleaved Caspase-3 (1:500; BM3257, Boster; Wuhan, China), TIMP2 (1:500; BM5024, Boster), Notch1 (1:1000; 20687-1-AP, Proteintech), TNF-α (1:1000; 60291-1-Ig, Proteintech), P53 (1:1000; 60283-2-Ig, Proteintech), Vimentin (1:1000; 60330-1-Ig, Proteintech), N-cadherin (1:1000; 66219-1-Ig, Proteintech), E-cadherin (1:1000; 60335-1-Ig, Proteintech) CD133 (1:1000; 18470-1-AP, Proteintech), Nestin (1:1000; 66259-1-Ig, Proteintech), Sox2 (1:1000; 11064-1-AP; Proteintech), LASS2 (1:1000; 20344-1-AP; Proteintech), and SPHK1 antibody (1:100; Proteintech) overnight at 4 °C. After washing 3 times with TBST for 5 min at room temperature, horseradish peroxidase (HRP)-conjugated goat anti-mouse (BA1051, Boster) and goat anti-rabbit (BA1055, Boster) secondary antibodies (both 1:1000; Boster) diluted in TBST containing 5% BSA were employed, followed by 3 washes with TBST (5 min each) at room temperature. Signal intensity measurement was performed with ImageJ software (NIH), and values were calculated as the average density multiplied by the area (measured in pixels).

### H&E staining and immunohistochemistry

Tumor tissues were cryosectioned at a 4 μm thickness for H&E staining based on previous work 10. Immunohistochemical staining was employed to evaluate the morphological expression and localization of LASS2, TIMP2, MMP9, CD133, Sox2, and Nestin. Deparaffinized sections were rehydrated through a graded series of ethanol and finally in PBS. After antigen retrieval, tissue samples were incubated in 3% H_2_O_2_ to quench endogenous peroxidase activity. Sections were then blocked with 10% normal goat serum for 30 min. Then, sections were individually incubated with rabbit anti-LASS2 (1:100; 20344-1-AP; Proteintech), anti-TIMP2 (1:100; 17353-1-AP; Proteintech), anti-MMP9 (1:100; 10375-2-AP; Proteintech), and SPHK1 antibody (1:100; Proteintech) antibodies overnight at 4 °C. Antigen-antibody complexes were visualized using the AEC method (Zhongshan Goldbridge Biotechnology Co., Ltd., Beijing, China). Tissues were then counterstained with hematoxylin (Zhongshan Goldbridge Biotechnology Co, Ltd.). Immunohistochemical staining was visualized under a Jiangnan light microscope (DN-10B, Jiangnan, Nanjing, Jiangsu).

### Statistical analysis

Statistical analyses were performed with SPSS (Statistical Package for the Social Sciences) 19.0 software (SPSS, Chicago, IL, USA). The data are expressed as the mean ± SEM of 3 to 5 independent experiments and were analyzed using either unpaired two-tailed independent Student's t-test or one-way ANOVA with Tukey's post hoc test for multiple comparisons. Differences were considered to be significant at P < 0.05.

## Results

### Evaluation of LASS2 immunostaining and LASS2/CD133 double immunostaining intensities in the microarray containing human glioma samples of different grades

**Figure 1:**
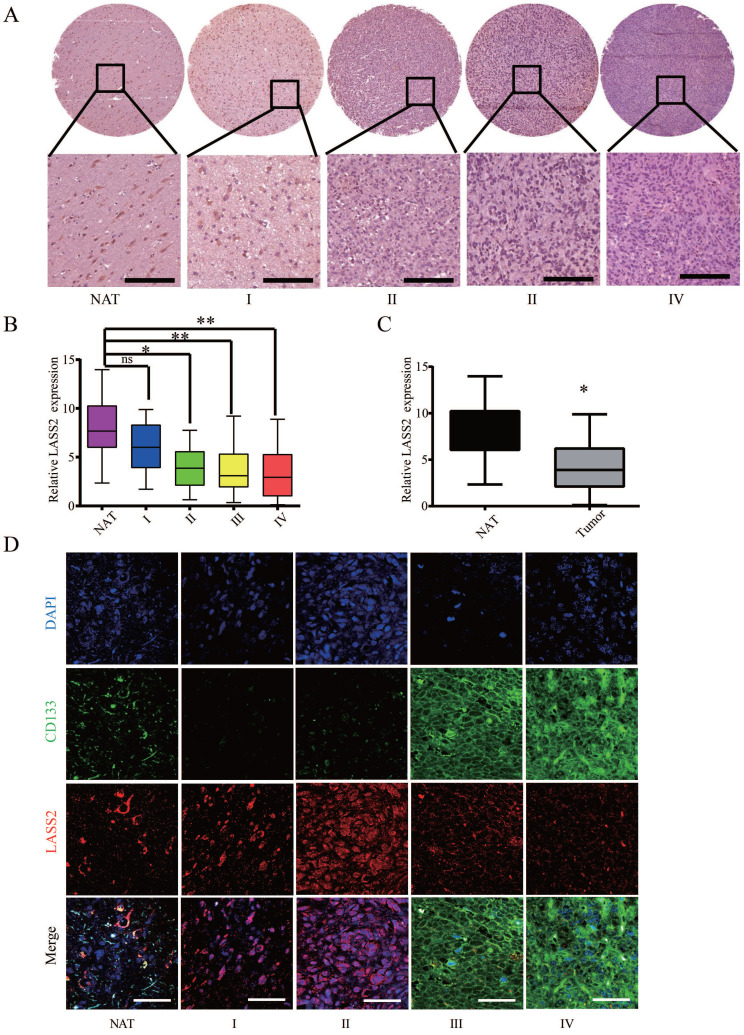
** Analysis of LASS2 expression on a human glioma tissue microarry. (A)** Representative immunohistochemical staining images of LASS2. Scale bar = 100 µm. **(B)** LASS2 levels in grade II to IV glioma samples were significantly lower than those in the normal adjancent tissue (NAT) (*P < 0.05 for grade II, and **P < 0.01 for both grade III and IV, n.s., no significance; one-way ANOVA). **(C)** The level of LASS2 in glioma of all grades was significantly lower than in NAT (*P < 0.05; unpaired two-tailed Student's t-test). **(D)** Representative images showing the immunuofluorescent co-staining of LASS2 and CD133 in the NAT and gliomas graded from I to IV. Scale bar = 20 µm.

Eight NAT, 30 grade I, 23 grade II, 25 grade III, and 35 grade IV tissue points were included for the immunohistochemical assay. Representative images of immunohistochemical staining of LASS2 in each group are shown in Fig. 1A. The protein level was measured as the integrated immunostaining intensity based on grayscale values ranging from 0-255 obtained using Image Tool II software. The integrated intensities of LASS2 immunostaining in grade I to IV gliomas decreased with increasing glioma malignancy grade (Fig. 1B). The LASS2 levels in grade II to IV glioma samples were significantly lower than those in the corresponding normal adjacent tissue (NAT) samples. However, there was no significant difference in the level of LASS2 between NAT and grade I samples (Fig. 1B). In total, the levels of LASS2 in glioma/glioblastoma tissues were significantly lower than those in NATs (Fig. 1C).

To investigate a possible relationship between LASS2 expression and the development of glioma stem cells, we performed double staining for CD133 and LASS2 in a glioma tissue microarray using an immunofluorescence approach. Our results demonstrated that both molecules were weakly detected at the NAT point. In grade I and II glioma tissues, the fluorescence signal of CD133 was rarely detected, whereas that of LASS2 was relatively high. In contrast, in grades III-IV tissues, the fluorescence signal of CD133 was strongly detected, whereas that of LASS2 was relatively low. The inverse correlation between LASS2 and CD133 demonstrated that LASS2 may be weakly expressed in GSCs derived from human glioma/glioblastoma tissues (Fig. 1D).

### Pathological characterization of CD133^+^ glioma stem cells

**Figure 2:**
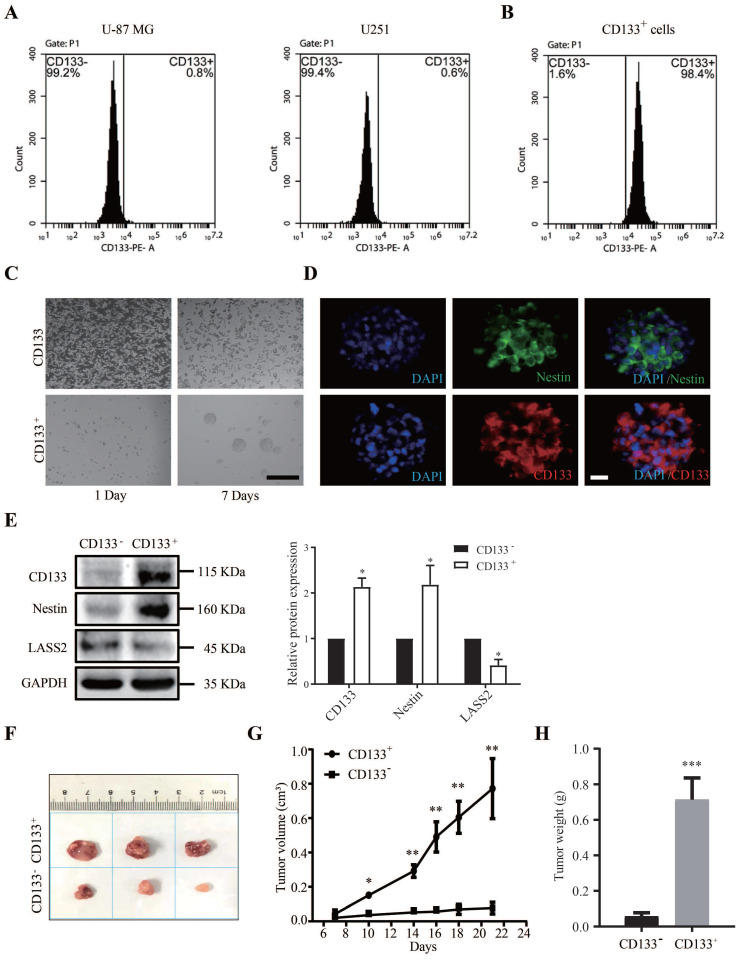
** Isolation of GSCs from U-87 MG and U251 cells. (A)** The proportion of CD133^+^ cells in U-87 MG and U251 cells. **(B)** The sorted CD133^+^ U-87 MG cells were retested by flow cytometry after 7 days of culture, and the percentages of CD133^+^ cells and CD133^-^ cells were shown. **(C)** CD133^+^ cells and CD133^-^ cells were maintained in the serum-free condition for 7 days. CD133^+^ cells formed spheres, whereas CD133^-^ cells showed degradation. Scale bar = 200 µm. **(D)** The sphere derived from sorted CD133^+^ cells was shown to express stem cell markers CD133 and Nestin. Scale bar = 20 µm. **(E)** CD133 and Nestin were overexpressed in proliferated sorted CD133^+^ cells compared with CD133^-^ cells, whereas CD133^-^ cells overexpressed LASS2 compared with CD133^+^ cells (*P < 0.05, vs. CD133^-^ group; unpaired two-tailed Student's t-test; n = 3). **(F, G)** The glioma xenografts derived from CD133^+^ cells were more malignant than those derived from CD133- cells. **(H)** The average final tumor weight of the xenograft derived from CD133^+^ cells was significantly higher than that from CD133^-^ cells in nude mice (**F, G, and H,** *P < 0.05, **P < 0.01, and ***P < 0.001, vs. CD133^-^ group; unpaired two-tailed Student's t-test; n = 3 animals).

We first analyzed the pathological characteristics of CD133^+^ glioma stem cells. We isolated CD133^-^ cells and CD133^+^ stem cells from glioblastoma U-87 MG and U251 cells. Fluorescence-activated cell sorting (FACS) analysis demonstrated that the CD133^+^ cell fraction accounted for 0.8% and 0.6% of the total populations of U-87 MG and U251 cells, respectively (Fig. 2A). The sorting efficiency of the CD133^+^ and CD133^-^ populations was reevaluated after FACS sorting. The purity of CD133^+^ GSCs isolated from the population of proliferated sorted U-87 MG cells was greater than 98.4% (Fig. 2B). We then cultured CD133^+^ and CD133^-^ cells in serum-free conditioned culture medium for 7 days, at which time point CD133^+^ cells had aggregated and formed spheres, whereas CD133^-^ cells had undergone apoptosis-like changes (Fig. 2C). The immunofluorescence staining results demonstrated that the stem cell markers CD133 and Nestin were both abundantly expressed in cells composing neurospheres (Fig. 2D). Western blot analysis demonstrated that the CD133 and Nestin protein levels in CD133^+^ glioma stem cells were significantly higher than those in CD133^-^ glioma cells, whereas the protein level of LASS2 in CD133^+^ glioma stem cells was significantly lower than that in CD133^-^ glioma cells (Fig. 2E). To demonstrate the roles of CD133^+^ and CD133^-^ cells in glioma growth, we induced the growth of glioma xenografts using both CD133^+^ and CD133^-^ glioma cells in nude mice. The average size of the CD133^+^ cell-derived glioma xenografts was dramatically and significantly larger than that of the xenografts derived from CD133^-^ cells at days 7, 10, 14, 16, 18, and 21 post-injection (Fig. 2F, G). The final weights of CD133^+^ cell-derived xenografts were significantly higher than those of xenografts derived from CD133^-^ cells (Fig. 2H). In summary, our data suggest that CD133^+^ cells are more malignant than CD133^-^ cells.

### LASS2 inhibits malignant behaviors of glioma stem cells

**Figure 3:**
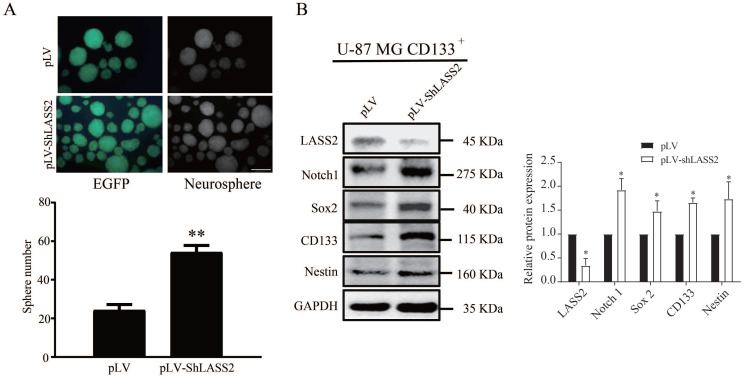
** LASS2 inhibits sphere formation of GSCs. CD133^+^ U-87 MG cells were stably transfected with pLV and pLV-*LASS2.* (A)** Downregulation of LASS2 promoted sphere formation of GSCs (**P < 0.01, vs. pLV control; unpaired two-tailed Student's t-test; n = 3). Scale bar = 200 µm. **(B)** Downregulation of LASS2 significantly reduced the protein level of LAAS2 while significantly increasing those of Notch1 and glioma stem cells stemness proteins CD133, Nestin and Sox2 (*P < 0.05, vs. pLV control; unpaired two-tailed Student's t-test; n = 3).

We employed sphere formation and western blot analyses to investigate the function of LASS2 in GSCs. The pLV-ShLASS2 treatment apparently promoted the sphere formation of GSCs in comparison to those treated with pLV (Fig. 3A). Compared with those treated with pLV control, treatment of GSCs with pLV-ShLASS2 significantly downregulated the protein level of LASS2 while significantly increasing those of Notch1 and GSCs stemness proteins Sox2, CD133, and Nestin (Fig. 3B).

### LASS2 functions as a tumor suppressor in glioblastoma cells

**Figure 4:**
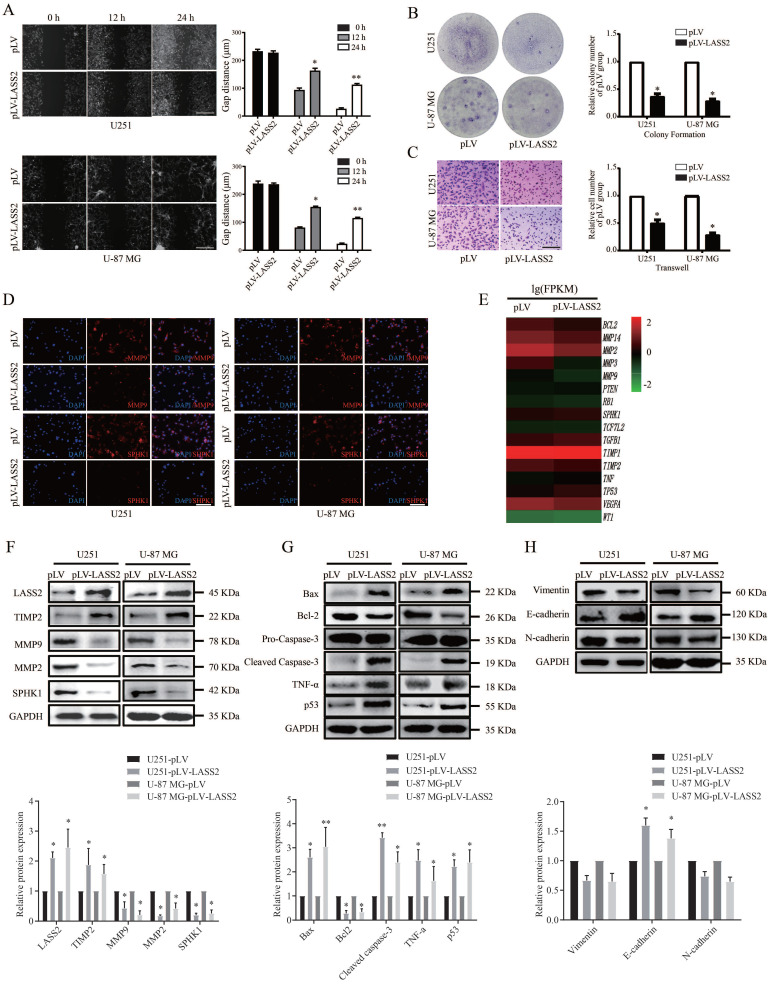
** Effect of LASS2 on cell migration, invasion and apoptosis. (A)** Wound healing assay of pLV-vector or pLV-LASS2-transfected glioma/glioblastoma cells at 0 h, 12 h and 24 h after scratch. The images were taken from an inverted microscope under 10× magnification (*P < 0.05 and **P < 0.01, vs. pLV control; unpaired two-tailed Student's t-test; n = 3). Scale bar = 200 µm. **(B)** Colony formation assay in pLV-vector or pLV-LASS2-transfected U251 and U-87 MG cells. Images were acquired at 4× magnification (*P <0.05, vs. pLV control; unpaired two-tailed Student's t-test; n = 3). **(C)** Transwell assay demonstrated that LASS2 inhibits the migration of U251 and U-87 MG cells compared with the pLV control (*P <0.05; unpaired two-tailed Student's t-test; n = 3). Scale bar = 200 µm. **(D)** The immunofluorescence staining of MMP9 and SPHK1 was shown in both U251 and U-87 MG cells. Scale bar = 200 µm. **(E)** RNA-Seq shows that LASS2 influenced cell migration/invasion, apoptosis, epithelial- mesenchymal transition (EMT) conversion and cellular life activity. **(F)** Overexpression of LASS2 reduced the protein levels of MMP2, MMP9, and SPHK1 while increasing that of TIMP2 in both U251 and U-87 MG cells. **(G)** LASS2 overexpression increased the levels of Bax, cleaved Caspase-3, TNF-α, and p53 while reducing that of Bcl-2 in both U251 and U-87 MG cells. **(H)** Overexpression of LASS2 reduced the protein levels of Vimentin and N-cadherin while increasing that of E-cadherin in both U251 and U-87 MG cells (F, G, and H, *P <0.05 and **P < 0.01, vs. pLV control in each cell line; unpaired two-tailed Student's t-test; n = 3).

To determine the functional roles of LASS2 in glioma/glioblastoma, we transfected either pLV-Vector or pLV-LASS2 plasmid into both U251 and U-87 MG cells. We then employed wound healing, colony formation, and transwell assays to determine whether LASS2 can modulate the migration and colony formation of glioma/glioblastoma cells. We found that *LASS2* overexpression significantly reduced the migration of U251 cells and U-87 MG cells (Fig. 4A) compared with that of empty scrambled control-treated cells. *LASS2* overexpression apparently inhibited the colony formation (Fig. 4B) and invasion (Fig. 4C) of both cell lines when compared with the corresponding empty scrambled control-treated cells. We also performed immunofluorescence staining assays to evaluate changes in MMP9 and SPHK1 levels in response to *LASS2* overexpression to evaluate cell migration and cell survival. The results demonstrated that *LASS2* overexpression significantly decreased the fluorescence signals of MMP9 and SPHK1 in both cell lines compared with the corresponding empty scrambled control-treated cells (Fig. 4D). Furthermore, we performed transcriptome sequencing of U-87 MG-pLV-LASS2 and U-87 MG-pLV cells. We found that LASS2 can regulate signaling molecules related to migration, invasion, epithelial-mesenchymal transition (EMT), and apoptosis in U-87 MG cells (Fig. 4E). The western blot analysis results demonstrated that overexpression of *LASS*2 significantly increased the protein level of TIMP2 while reducing those of MMP9, MMP2, and SPHK1 in both U251 and U-87 MG cells (Fig. 4F). In addition, overexpression of *LASS2* significantly increased the protein levels of Bax, cleaved Caspase-3, TNF-α, and p53 while reducing that of Bcl-2 in both cell lines (Fig. 4G). The western blot analysis results also demonstrated that overexpression of *LASS*2 reduced the protein levels of Vimentin and N-cadherin while significantly increasing that of E-cadherin in both U251 and U-87 MG cells (Fig. 4H).

### LASS2 inhibits glioma growth *in vivo* in a nude mouse model

**Figure 5:**
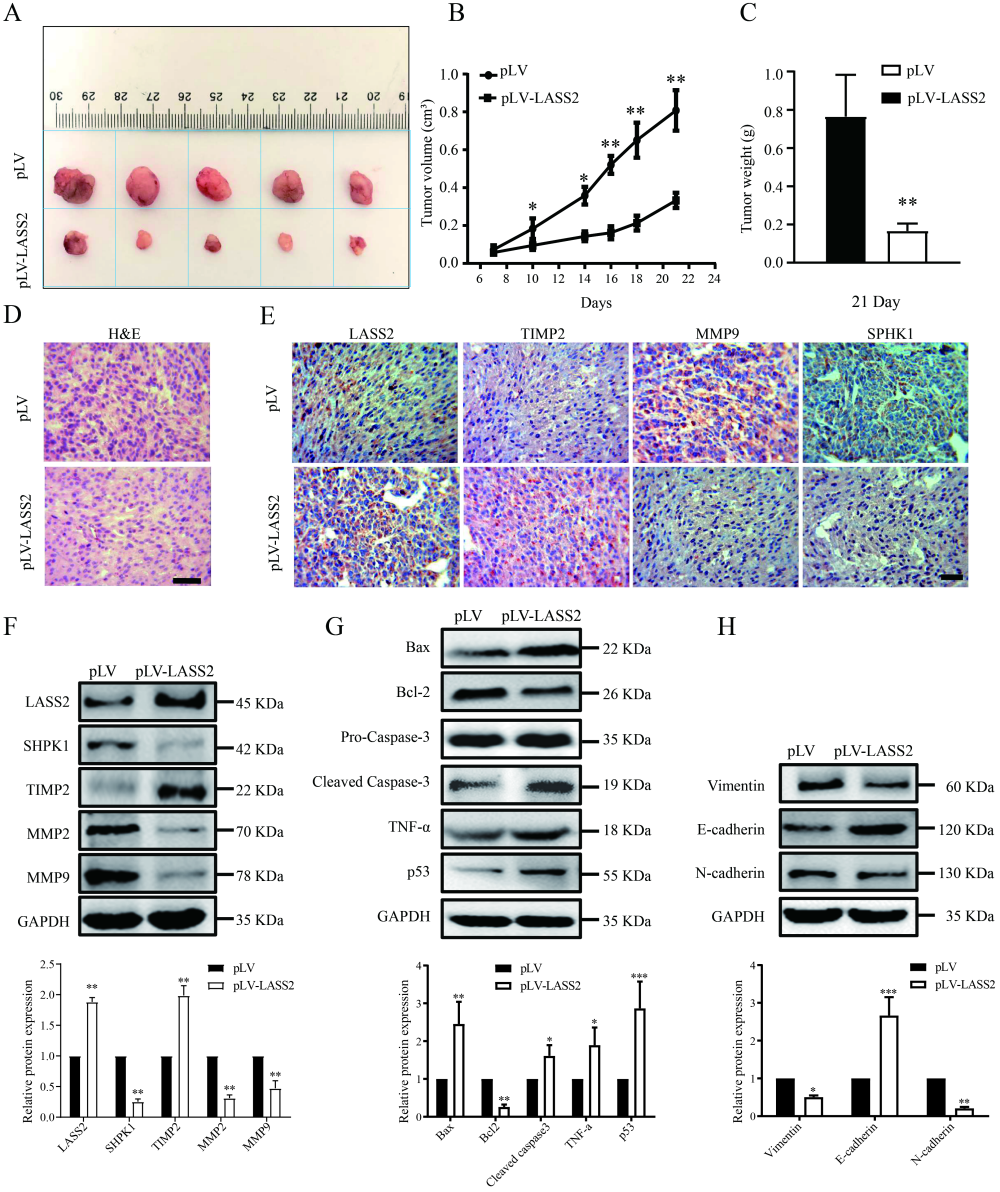
** LASS2 inhibited tumor growth in a pLV-LASS2-U-87 MG glioblastoma xenograft nude mouse model. (A)** Representative photographs showing the gross pLV-LASS2-U-87 MG and empty scramble control glioblastoma xenografts from the nude mouse. **(B)** The tumor volume was evaluated between the scrambled control and pLV-LASS2 groups (**P < 0.05 and **P < 0.01 vs. pLV control group; unpaired two-tailed Student's t-test; n = 5 animals). **(C)** The final tumor weight was measured after dissection. The average final weight of tumors derived from pLV-LASS2-transcfected U-87 MG cells was significantly lower than those derived from the scrambled control (**P < 0.01, vs. pLV control group; unpaired two-tailed Student's t-test; n = 5 animals). **(D)** Representative images for H&E staining from either group were shown. **(E)** IHC staining of LASS2, TIMP2, MMP9, and SPHK1 in xenografted tumors derived from U-87 MG cells transfected with either pLV-LASS2 or scrambled control. Scale bar = 20 µm. **(F)** Western blot analysis of LASS2, SPHK1, TIMP2, MMP2, and MMP9 in xenografted tumors derived from U-87 MG cells transfected with either pLV or pLV-LASS2. **(G)** Western blot analysis of Bax, Bcl-2, pro-Caspase-3, cleaved Caspase-3, TNF-α and p53 in xenografted tumors derived from U-87 MG cells transfected with either pLV or pLV-LASS2. **(H)** Western blot analysis of EMT conversion-related proteins Vimentin, E-cadherin, and N-cadherin in xenografted tumors derived from U-87 MG cells transfected with either pLV or pLV-LASS2 (**F, G, and H,** *P <0.05, **P < 0.01, and ***P < 0.001, vs. pLV control; unpaired two-tailed Student's t-test; n = 3).

To determine the role of LASS2 in modulating glioma growth, we established xenografts using U-87 MG cells stably transfected with either pLV-vector or pLV-LASS2 plasmid. Excised xenografts are shown in Fig. 5A and indicate that *LASS2* overexpression inhibited tumor growth at all time points tested until treatment completion (Fig. 5B). The average final weight of glioma tumors with *LASS2* overexpression was significantly lower than that of glioma tumors with vector overexpression at day 21 (Fig. 5C). H&E staining showed a relatively less dense structure in tumor tissue from the U-87 MG-pLV-LASS2 group (Fig. 5D). Immunohistochemical staining also demonstrated that MMP9 and SPHK1 levels were apparently reduced, but LASS2 and TIMP2 levels were increased in glioma xenografts derived from U-87 MG-pLV-LASS2 cells compared with those derived from U-87 MG-pLV cells (Fig. 5E). Western blot analysis demonstrated that the protein levels of LASS2 and TIMP2 were apparently elevated, accompanied by reductions in SPHK1, MMP2, and MMP9 protein levels, in glioma xenografts derived from U-87 MG-pLV-LASS2 cells compared with those derived from U-87 MG-pLV cells (Fig. 5F). Western blot analysis demonstrated a reduction in the protein level of Bcl-2 and an increase in the protein levels of Bax, cleaved Caspase-3, TNF-α, and p53 in glioma xenografts derived from U-87 MG-pLV-LASS2 cells compared with those derived from U-87 MG-pLV cells (Fig. 5G). Western blot analysis also demonstrated that the protein levels of Vimentin and N-cadherin were reduced, but that of E-cadherin was increased in glioma xenografts derived from U-87 MG-pLV-LASS2 cells compared with those derived from U-87 MG-pLV cells (Fig. 5H).

## Discussion

So far, glioma is still one of the critical threats to human health. The genesis and development of glioma is a multi-process, including astrocyte, stem cell proliferation, tumor proliferation, and EMT, etc. LASS2 is one of the ceramide synthases. Accumulated evidence indicates that LASS2 is involved in cancer progression and tumor chemosensitivity 11, mainly including bladder cancer 12, hepatoblastoma 13, and prostate cancer 14. However, the role of LASS2 in glioma remains elusive. This study investigated the role of LASS2 in GSCs proliferation and sphere formation. The role of LASS2 in glioma/glioblastoma cell migration/invasion and tumor formation was also investigated.

Previous studies have suggested that LASS2 inhibits cancer development through inhibition of the vacuolar-H (+)-ATPase, which functions mainly by controlling the acidification of internal and external cells under physiological and pathological conditions 15. Some V-ATPase subunits were found to be enriched in glioma stem cells and in patients with poor survival outcomes 16, which promotes us to explore the role of LASS2 in GSCs.

GSCs are capable of self-renewal, thus replenishing a glioma cell population with metastatic growth characteristics. The chemo- and radio-resistance of GSCs greatly contribute to the tumor response to anticancer treatment 17. The GSC hypothesis has been revived based on the discovery of marker profiles for prospective identification of GSCs, which has led to the identification of novel treatments specifically targeting GSCs. Thus, further understanding of the mechanisms underlying GSCs proliferation may facilitate our identification of a promising method for the treatment of glioma and improve future anticancer therapies. It was noted that *LASS2* overexpression in CD133^+^ U-87 MG cells inhibited GSC sphere formation and reduced the protein levels of Notch1, CD133, Nestin, and Sox2, thus implying that *LASS2* can influence glioma stem cell-specific proteins and inhibit stemness maintenance of GSCs, and thus reduce the growth of GSCs-derived glioblastoma growth.

Recently, LASS2 has been reported to modulate tumor cell growth via different cell signaling in a tumor type-dependent manner. LASS2 inhibits bladder cancer invasion and chemoresistance through regulation of ERK-Drp1 induced mitochondrial dynamics 18. In addition, LASS2 inhibits growth and invasion of bladder cancer by regulating ATPase activity, suggesting that LASS2 silencing may enhance the growth, invasion, and metastasis of cancer cells by regulating ATPase activity 12. LASS2 inhibits proliferation and induces apoptosis in HepG2 hepatoblastoma cells through the mitochondrial apoptotic, NF‑κB, and cell cycle signaling pathways 13. Phosphorylated LASS2 also inhibits prostate carcinogenesis through negative regulation of Wnt/β-catenin signaling 14.

Epithelial-mesenchymal transition is a highly conserved step-wise process, which refers to the transition of migratory mesenchymal cells from immobile epithelial cells with the loss of tight junctions and associated adherence 19. E-cadherin is a transmembrane glycoprotein that exerts a tumor-suppressing role in normal cells, whose down-regulation usually indicates poor prognosis and survival in patients of various cancers 20. In contrast to the loss of E-cadherin functions during EMT, Vimentin and N-cadherin are up-regulated as mesenchymal markers to enhance cells to migrate or metastasize to target organs 21. It has been reported that overexpression of integrin-linked kinase (ILK) can down-regulate E-cadherin in an NF-κB pathway-dependent manner in glioma cells with enhanced invasion and migration potentials 22. The enhanced N-cadherin expression has been shown to compound the EMT process in gliomas with unfavorable prognostic outcomes, although no correlated overall survival has been detected 23. We here demonstrated that *LASS2* overexpression significantly down-regulated the expression of N-cadherin while increasing that of E-cadherin both *in vitro* and *in vivo*, suggesting that LASS2 can function by inhibiting glioma/glioblastoma growth partly via the suppression of EMT.

In addition, LASS2 has been demonstrated to promote tumor cell apoptosis via ceramide 24, 25, an effect that can be reduced when ceramide is converted into survival-promoting sphingosine 1-phosphate (S1P) by sphingosine kinase 1 (SPHK1) in glioblastoma 26, 27. Overexpression of *LASS2* reduced the protein expression of SPHK1, MMP9, MMP2, Bcl-2, Vimentin, and N-cadherin while increasing that of TIMP2, Bax, activated caspase3, TNF-α, p53, and E-cadherin both *in vitro* and *in vivo*, suggesting that LASS2 suppresses the development and progression of glioma mainly by inhibiting tumor cell migration, invasion, and proliferation 28-30. The inhibitory effect of LASS2 on subcutaneous xenograft growth further supports previous reports that *LASS2* can function as a novel tumor-suppressing gene in glioma formation, progression, and metastasis.

However, high-leveled LASS2 was reported in endometrial cancer tissues, where it contributes to progestin resistance under the modulation of Nrf2 31. Increased expression of LASS2 plays an unfavorable role in the prognosis in patients with ovarian cancer by enhancing migration, invasion, and metastasis of cancer cells 32. Thus, further efforts toward the development of LASS2-based glioma treatment are warranted.

## Conclusions

In summary, our findings indicate that LASS2 can function as a suppressor of glioma growth, suggesting that modulation of *LASS2* expression may contribute to a novel strategy for the management of glioma via inhibition of GSCs. Further investigation on LASS2 may provide a better understanding of glioma/glioblastoma pathogenesis and may ultimately promote the development of efficient therapies based on the promotion of LASS2 function.
